# Machine Learning Algorithms to Estimate Propensity Scores in Health Policy Evaluation: A Scoping Review

**DOI:** 10.3390/ijerph21111484

**Published:** 2024-11-07

**Authors:** Luís Lourenço, Luciano Weber, Leandro Garcia, Vinicius Ramos, João Souza

**Affiliations:** 1 Department of Knowledge Engineering, Federal University of Santa Catarina, Florianópolis 88035-972, Brazilv.ramos@ufsc.br (V.R.);; 2 Piccolo Mental Health, Florianópolis 88035-400, Brazil; lpgarcia18@gmail.com

**Keywords:** causality, artificial intelligence, health care economics and organizations

## Abstract

(1) Background: Quasi-experimental design has been widely used in causal inference for health policy impact evaluation. However, due to the non-randomized treatment used, there is great potential for bias in the assessment of the results, which can be reduced by using propensity score (PS) methods. In this context, this article aims to map the literature concerning the use of machine learning (ML) algorithms for propensity score estimation. (2) Methods: A scoping review was carried out in the PubMed, EMBASE, ACM Digital Library, IEEE Explore, LILACS, Web of Science, Scopus, Compendex, and gray literature (ProQuest and Google Scholar) databases, based on the PRISMA-ScR guidelines. This scoping review aims to identify ML models and their accuracy and the characteristics of studies on causal inference for health policy impacts, with a specific focus on PS estimation using ML. (3) Results: Seven studies were included in the review from 3018 references searched. In general, tree-based ML models were used for PS estimation. Most of the studies did not show or mention the performance metrics of the selected models, focusing instead on discussing the treatment effects under analysis. (4) Conclusions: Despite important aspects of model development and evaluation being under-reported, this scoping review provides insights into the recent use of ML algorithms in health policy impact evaluation.

## 1. Introduction

Causal inference is a statistical method that aims to provide evidence about the impact of an intervention on a system, and it has been widely employed in empirical research to assess the evaluation of public health policies [1]. In the case of public health policy impact evaluation, the core of causal inference is to provide a reliable answer to the following question: “What would have happened in the absence of the policy?”. This question represents the counterfactual scenario (contrary to the fact), which cannot be directly observed [2]. Therefore, it is necessary to estimate the potential outcomes in the situation where the policy had not been implemented, by comparing a group of individuals subject to the policy (treatment group) with another group that is not subject to it (control group). If the selected groups have similar characteristics, it is possible to estimate the policy impact effect without bias [3,4,5].

Overall, researchers use data from quasi-experimental design research (ecological studies) to investigate the causal effect of public health policies. One of the challenges of causal inference in ecological studies is the presence of imbalances in covariates, which affect both the treatment and the outcome, acting as confounders [6]. In this context, a set of assumptions is required to obtain unbiased causal inference: stability (the potential outcomes for any unit are not affected by other units’ treatment levels), consistency (the observed outcome is equal to the potential outcome at any treatment level), exchangeability (the potential outcomes are independent of the treatment for any treatment level), and positivity (subgroups with similar characteristics have a non-zero probability of being in any intervention level) [7].

However, the large number of covariates and the non-randomized nature of subject groups in quasi-experimental studies pose significant challenges to causal inference [8]. Confounding factors, selection bias, and measurement errors are typically bottlenecks in validating causal inference models [9]. To overcome these issues, propensity score (PS)-based methods are often used to reduce bias in causal effect estimation [4]. PS is a conditional probability of receiving a specific treatment (or an intervention, e.g., the implementation of a health policy) in a quasi-experimental design. Additionally, PS incorporates observed covariables potentially related to the treatment under analysis and/or its outcomes in a single score [10,11].

The PS approach results in a dimensionality reduction, since a single variable is obtained from a given vector of observed covariables [12]. Thus, all covariates are incorporated into the PS, offering a way to achieve more balanced groups through the PS matching of the treatment and control groups. Causal inference can then be made by balancing on the PS rather than on all the covariates. Finally, given an exposed and an unexposed individual with a similar PS, the assignment of treatment is independent of unobserved confounders [8]. In the extended literature, logistic regression has been broadly employed to estimate the PS [12,13,14,15]. However, logistic regression requires assumptions regarding variable selection, the distributions of variables, and the specification of interactions [16]. In this context, machine learning (ML) algorithms are an alternative to logistic regression in estimating the PS [17].

ML stands as a prominent application of artificial intelligence (AI) and has been used to obtain insights in different fields. In public health, several ML algorithms have been employed to predict clinical outcomes using data from medical imaging and electronic health records (EHRs), for instance [18]. However, despite the widespread use of predictive ML models in clinical medicine, the application of AI to assess the impact of public health policies on population health and health inequalities has been less reported [2,6,19].

This scoping review aims to map the empirical literature concerning the use of ML algorithms for PS estimation in the causal inference evaluation of public health policies. Our goal is to identify ML algorithms that can be applied to estimate the PS, as well as the modeling and evaluation strategies involved. The key contribution of this study is to provide a comprehensive overview of the potential use of ML techniques in PS estimation and their applicability to real-world public health data.

To our knowledge, few reviews have focused specifically on the use of ML in causal inference related to public health policy, differing from this scoping review in terms of their objectives and scope. The review conducted by Westreich et al. (2010) [12] explores ML alternatives to logistic regression, primarily offering theoretical explanations of the models. Mooney and Pejaver (2018) [9] address several key issues around big data in public health, including a brief discussion on causal inference. Cheng et al. (2022) [17] focus on reviewing causal theories and methodologies, and on benchmarking a fundamental task in causal inference.

In this scoping review, we provide an analysis of the use of ML algorithms to estimate the PS in health policy evaluation in empirical studies. Moreover, understanding how these models have been developed, identifying knowledge gaps, and recognizing limitations might guide future research in causal inference, providing important contributions towards a better understanding of causal inference powered by ML algorithms.

## 2. Materials and Methods

This scoping review follows the Preferred Reporting Items for Systematic Reviews and Meta-Analyses extension for Scoping Reviews (PRISMA-ScR) guidelines [20], and the study protocol was registered on the Open Science Framework (doi.org/10.17605/OSF.IO/WTMGX).

### 2.1. Research Question

The research question was developed using the PCC (Participants, Concept, and Context) framework. The context involves the evaluation of public health policies, while the concept focuses on the use of machine learning (ML) algorithms for propensity score (PS) estimation. The participants are undefined and refer to the general population under a specific health policy. Thus, the research question is as follows: *“What machine learning algorithms have been used to estimate propensity scores in the context of health policy impact evaluation?”*. Our goal is to comprehensively scope out all ML algorithms that have been employed in causal inference for evaluating health policies worldwide.

### 2.2. Eligibility Criteria

The inclusion criteria covered all articles that use any ML algorithm to estimate the PS in the context of health policy impact evaluation. There were no restrictions on the publication year, language, or location. The exclusion criteria were as follows: (1) studies outside the domain of public policy evaluation; (2) studies that did not employ any ML algorithm to estimate PS; (3) studies focused on a specific clinical care or disease application; (4) those with missing methodology or results; (5) studies comprising book chapters or conference papers; and (6) studies where the full text was not available.

### 2.3. Information Sources and Search Strategy

The search strategy comprised peer-reviewed literature database, gray literature, and reference searches. The following databases were selected: PubMed, EMBASE, ACM Digital Library, IEEE Explore, LILACS, Web of Science, Scopus, and Compendex. Gray literature search tools included ProQuest and Google Scholar. The Google Scholar search was based on the first 100 most relevant studies. The search strategy was developed in collaboration with an academic librarian and adapted for each database (Appendix A). The search was conducted in 6 July 2023. Additionally, the reference lists of included articles were screened to identify potential studies. The references obtained from the search strategy were managed using EndNote Web, which automatically removed duplicate articles.

### 2.4. Study Selection Process

The study selection process and decision tracking were managed using the Rayyan review tool. Two independent reviewers (LL and LW) conducted the screening in a double-blinded process, following two sequential phases. In the first phase, the selection process was based on reading the title, abstract, and author keywords. Studies which met the eligibility criteria were selected and moved to the next phase. In the second phase, full-text reading was undertaken, applying the inclusion and exclusion criteria to obtain the final set of studies (phase 2). In both phases, disagreements were resolved through consensus, and in cases of persisting discrepancies, a third reviewer (VR) was consulted.

### 2.5. Data Extraction and Synthesis

Data from each included study were extracted independently by two reviewers (LL and LW). The extracted data included general study characteristics (country, health policy or program under analysis, year, and data source), model development (features, outcomes, and ML algorithms used) and evaluation aspects (performance metrics and main conclusions). The data charting process was conducted manually. In order to minimize potential errors, a calibration study was carried out as indicated in the PRISMA-ScR guidelines [20]. Disagreements were resolved through consensus. The results were synthesized through a narrative approach, reporting on the extent of different ML models for PS estimation in causal impact of health policies, the methods employed, and whether bias was identified.

## 3. Results

### 3.1. Study Selection

**Figure 1:**
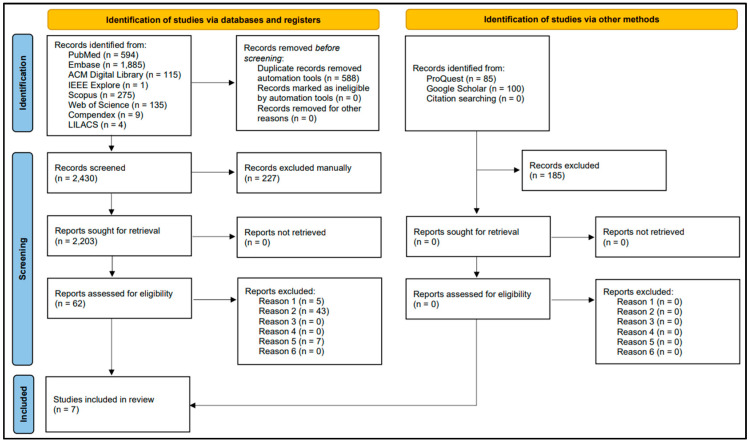
PRISMA flow diagram of literature search and selection criteria.

The search retrieved 3018 studies from the selected database and 185 studies from the gray literature. A total of 815 duplicated articles were automatically excluded, leaving 2203 documents remaining. After screening phase 1, based on reading the title, abstract, and author keywords, 2141 studies were excluded. In screening phase 2, 55 articles were excluded and 7 studies met the inclusion criteria after full-text reading. The screening process is presented in Figure 1, and data extracted from all the included studies are summarized in Table 1.

### 3.2. Machine Learning Algorithms

The ML algorithms used in the screened studies included random forest [22,24,27], causal forest [21,25], Bayesian additive regression trees [22,26], neural networks [24,27], gradient boosting [24,27], generalized linear models [27], XGBoost [27], boosted regression trees [23], and support vector machines [24]. In addition, three studies compared the results of the machine learning algorithms with statistical logistic regression methods [22,24,25].

All the studies reviewed provided some discussion regarding the use of ML algorithms for assessing the impact of health policies. The main advantages highlighted by the authors included efficiency in handling high-dimensional data, the ability to capture the non-linear relationships among the variables, improved covariate balance, and reduction in bias in cases of model misspecification. The authors of most of the articles also discussed some of the limitations of their studies.

### 3.3. Model Development

In general, sociodemographic variables such as age, gender, education level, and household income, were frequently used. The number of features considered in each study varied between 5 and 33, with 57% of the studies using less than 10 independent variables. Fewer authors described how missing data were handled [22,27], reported how overfitting was prevented [24], provided the algorithm’s hyperparameters [23,25], and evaluated performance metrics [24,27]. Additionally, a sensitivity analysis was performed and evaluated only in three selected studies [22,24,27]. Sensitivity analysis approaches are recommended in causality studies to assess the robustness of causal effects in the presence of potential unmeasured or uncontrolled confounders [28,29].

## 4. Discussion

This scoping review provides a comprehensive overview of the current use of ML algorithms for PS estimation in health policy impact evaluation studies worldwide. A total of seven studies were included in the review process, evidencing the incipient application of ML for this purpose. Despite the growing presence of artificial intelligence in health sciences [1,9], it is well known that statistical models are widely employed to estimate propensity scores in causal inference studies. Among them, logistic regression is preferrable due to its familiarity among researchers and ease of implementation, often being considered the state-of-the-art model [12].

In their study, Garcia et al. (2023) [27] proposed the use of ML to estimate the impact of national government expenditure on reducing infant mortality across several countries. Although the study does not focus on a specific health policy, the authors sought to investigate the decisions made by governments to improve public health in their countries. The method employed was based on the use of the Super Learner algorithm to estimate the generalized propensity score (GPS), guiding the results to a reduction in the coefficient of variation of the weights. The authors compared the results with other GPS estimation methods and found that Super Learner had the highest coefficient of variation of weights. Moreover, specific performance metrics of Super Learner were not provided, making it impossible to assess the feasibility of this approach.

Bayesian additive regression trees (BARTs) and boosted regression trees were used by Wink Junior et al. (2022) [26] and Ladhania et al. (2021) [23], respectively, to evaluate the impact of health policies on emergency care units. Although both studies discussed the potential advantages of ML in causal inference, the authors did not provide a detailed evaluation of the selected models. Likewise, a causal forest was applied in two screened studies [21,25]. However, both analyses did not show or mention any performance metrics of the models, and also did not perform a sensitivity analysis.

ML applications can be particularly effective in modeling the association of outcomes with covariates, especially when dealing with high-dimensional data [2]. In addition, ML algorithms can implicitly address interactions and non-linearities, which might result in improvements in PS estimation, as logistic regression assumes linearity between covariates and the log odds of the outcome. When there are implicit non-linearities in the system, the use of logistic regression might result in a poor model fit and, in turn, a biased effect estimation [12,16]. In this context, Westreich et al. (2010) [12] discussed the use of ML for PS estimation, highlighting boosting techniques and decision trees as alternatives to logistic regression. However, the main drawback of these algorithms is their “black box” nature, which can make the etiological interpretation difficult.

Lee, Lessler, and Stuart (2010) [16] explored the use of ML algorithms to estimate the PS in a hypothetical study in order to evaluate the robustness of non-parametric techniques compared to logistic regression. The authors evaluated the performance of basic off-the-shelf versions of ML, reducing the complexity of implementation, across scenarios with different degrees of non-linearity as well as using small, medium, and large-size datasets. According to their findings, the classification and regression trees (CARTs), random forests, and boosted CARTs outperformed logistic regression, regardless of the sample size or the extent of the non-linearity. These results suggest that, despite ML algorithms usually being used with larger datasets, they can also be satisfactorily used in smaller datasets and less complex systems.

Two eligible studies analyzed different ML algorithms, providing comparison evaluations among the selected models and logistic regressions. Maciel and Duarte (2022) [24] compared the accuracy and sensitivity of gradient boosting, random forests, neural networks, support vector machines, and logistic regression models. According to their findings, all the models had a similar accuracy, ranging from 74.2% to 76.5%. However, gradient boosting had the highest sensitivity (93.5%), indicating a greater robustness to potential unobserved confounders [30]. On the other hand, both logistic regression and support vector machines had the lowest sensitivity (76.5%). Costello et al. (2021) [22] evaluated random forests and Bayesian additive regression trees, as well as logistic regression, for propensity score estimation. The authors support their results on the comparison of the estimation effects and the sensitivity analysis, regardless of them not providing appropriate ML performance metrics.

Overall, the findings suggest similar results among parametric and non-parametric models, demonstrating a relative robustness to unmeasured confounders. The satisfactory performance of logistic regression in both Maciel and Duarte’s (2022) [24] and Costello et al.’s (2021) [22] studies may argue against the use of ML algorithms for PS estimation for health policy evaluation. Considering the more demanding implementation of ML also in terms of computational time, Cannas and Arpino (2019) [6] pointed out that the use of ML over logistic regression may not be worthwhile if logistic regression is sufficient for achieving a good covariate balance.

The results of the selected studies often fell short in terms of model evaluation. Many studies did not report the hyperparameter selection process, strategies to avoid overfitting, or any performance metric, which limits their critical appraisal or comparison with parametric approaches. As a result, the discussion of the ML models is restricted to their use and application as an alternative to logistic regression. Consequently, the discussions on ML models were often restricted to their application as alternatives to logistic regression. This limitation hinders the comprehensive comparison of different ML algorithms used for PS estimation or the evaluation of different causal inference models across various studies.

Cheng et al. (2022) [17] highlighted the lack of benchmark resources for model evaluation in causal inference studies. This gap makes it challenging to integrate both ML and causal inference techniques. Moreover, tuning hyperparameters and validation processes are crucial to ensure reliable results from ML algorithms. In the context of causal inference, ML tuning is not straightforward, as optimality should refer to some measure of covariate balance required for unbiased effect estimation, rather than to goodness-of-fit measures, as is commonly performed [6]. Frequently, cross-validation techniques have been suggested for this purpose and they are recommended for future studies.

## 5. Conclusions and Future Directions

This scoping review acknowledges that some studies may have been omitted due the restrictions on the selected databases. In addition, the inclusion criteria were restricted to studies that specifically focused on health policy evaluation. Consequently, this review does not cover the broader use of machine learning in causal inference, potentially introducing selection bias. Since many studies did not report the performance metrics of the algorithms, this review primarily aimed to discuss the state of the art of the use of machine learning for propensity score estimation, rather than providing a comprehensive evaluation of its feasibility and advantages compared to parametric methods. Despite these challenges, the results of this scoping review highlighted key directions for future research. When dealing with high-dimensional covariates, a parametric approach such as logistic regression may fail to address the interaction effects of all the predictors. In this case, ML algorithms can offer an alternative to overcome this issue. However, if the system under analysis involves few covariates, it is necessary to evaluate whether the use of ML algorithms over logistic regression, for instance, brings significant benefits, due to the model complexity and computational costs. In summary, while the application of ML algorithms remains limited, there is clear potential for further exploration. More primary studies are needed to better understand the precision of AI in health policy evaluation. Overall, ML offers promising opportunities to enhance the evaluation of health policies by incorporating high-dimensional data and non-linear effects, thus enriching our understanding of policy interventions.

## Figures and Tables

**Table 1:** Data summary of included studies on machine learning used for propensity score estimation.

Author, Year, Country	Health Policy/Program	Data SOURCE	ML Algorithms	Performance Metrics
Chen et al., 2021 China [21]	Urban and Rural Resident Basic Medical Insurance (URRBMI)	China Family Panel Studies (CFPS)	Causal forest	Not provided
Costello et al., 2021 Canada [22]	12-step groups	Recovery Journey Project (RJP)	Random forest; Bayesian additive regression trees (BARTs)	Not provided
Ladhania et al., 2021 USA [23]	Medicaid	National Hospital Ambulatory Medical Care Survey	Boosted regression trees	Not provided
Maciel and Duarte, 2022 USA [24]	Cash transfer program Bolsa Família	Brazilian Household Budget Survey	Gradient boosting; random forest; neural networks; SVM	Accuracy (%); misclassification rate (%)
Kreif et al., 2022 UK [25]	Jamima Kesehatan National (JKN)	Indonesian Family Life Survey	Causal forest	Not provided
Wink Junior et al., 2022 Brazil [26]	National Policy for Emergency and Urgent Care	DATASUS; Brazilian Institute of Geography and Statistics; National Supplementary Health Agency	Bayesian additive regression trees (BARTs)	Not provided
Garcia et al., 2023 Brazil [27]	Public expenditure on health per capita	World Bank database	SuperLearner (generalized linear model, neural network, random forest, gradient boosting machine, Xgboost)	Not provided

## Appendix A. Search Strategy in Each Database

Database: PubMed/MEDLINE

Search query:

(“Machine Learning” [MeSH Terms] OR “Machine Learning” [All Fields] OR “Transfer Learning” [All Fields] OR “Artificial Intelligence” [MeSH Terms] OR “Artificial Intelligence” [All Fields] OR “Deep Learning” [MeSH Terms] OR “Deep Learning” [All Fields] OR “cart” [Title/Abstract] OR “classification and regression tree” [All Fields] OR “gradient boosting machine” [All Fields] OR “xgboost” [All Fields] OR “neural networks, computer” [MeSH Terms] OR “Computer Neural Network” [All Fields] OR “Computer Neural Networks” [All Fields] OR “Neural Networks” [All Fields] OR “Neural Network” [All Fields] OR “Random Forest” [MeSH Terms] OR “Random Forest” [All Fields] OR “Random Forests” [All Fields] OR “logistic regression” [All Fields] OR “decision tree” [All Fields]) AND (“Health Policy” [MeSH Terms] OR “Health Policy” [Title/Abstract] OR “Healthcare Policy” [All Fields] OR “Healthcare Policies” [All Fields] OR “Health Policies” [All Fields] OR “Health Care Policies” [All Fields] OR “Health Care Policy” [All Fields] OR “National Health Policy” [All Fields] OR “National Health Policies” [All Fields] OR “public health” [Title/Abstract] OR “Health Care Economics and Organizations” [MeSH Terms] OR “Healthcare Economics and Organizations” [All Fields] OR “Health Care Economics” [All Fields] OR “Health Care Economic” [All Fields] OR “Healthcare Economics” [All Fields] OR “Healthcare Economic” [All Fields] OR “Health Economics” [All Fields] OR “Health Economic” [All Fields]) AND (“Propensity Score” [MeSH Terms] OR “Propensity Score” [All Fields] OR “Propensity Scores” [All Fields] OR “Inverse Propensity Weighting” [All Fields])

Database: Embase (Elsevier)

Search query:

(‘machine learning’/de OR ‘machine learning’ OR ‘transfer learning’/de OR ‘transfer learning’ OR ‘artificial intelligence’/de OR ‘artificial intelligence’ OR ‘deep learning’/de OR ‘deep learning’ OR ‘cart’ OR ‘classification and regression tree’/de OR ‘classification and regression tree’ OR ‘gradient boosting machine’/de OR ‘gradient boosting machine’ OR ‘xgboost’/de OR ‘xgboost’ OR ‘computer neural network’/de OR ‘computer neural network’ OR ‘computer neural networks’/de OR ‘computer neural networks’ OR ‘neural networks’/de OR ‘neural networks’ OR ‘neural network’/de OR ‘neural network’ OR ‘random forest’/de OR ‘random forest’ OR ‘random forests’/de OR ‘random forests’ OR ‘logistic regression’/de OR ‘logistic regression’ OR ‘decision tree’/de OR ‘decision tree’) AND (‘health policy’/de OR ‘health policy’ OR ‘healthcare policy’/de OR ‘healthcare policy’ OR ‘healthcare policies’ OR ‘health policies’ OR ‘health care policies’ OR ‘health care policy’/de OR ‘health care policy’ OR ‘national health policy’ OR ‘national health policies’ OR ‘public health’/de OR ‘public health’ OR ‘healthcare economics and organizations’ OR ‘health care economics’/de OR ‘health care economics’ OR ‘health care economic’ OR ‘healthcare economics’/de OR ‘healthcare economics’ OR ‘healthcare economic’ OR ‘health economics’/de OR ‘health economics’ OR ‘health economic’) AND (‘propensity score’/de OR ‘propensity score’ OR ‘propensity scores’/de OR ‘propensity scores’ OR ‘inverse propensity weighting’)

Database: ACM

Search query:

(“Machine Learning” OR “Transfer Learning” OR “Artificial Intelligence” OR “Deep Learning” OR “cart” OR “classification and regression tree” OR “gradient boosting machine” OR “xgboost” OR “Computer Neural Network” OR “Computer Neural Networks” OR “Neural Networks” OR “Neural Network” OR “Random Forest” OR “Random Forests” OR “logistic regression” OR “decision tree”) AND (“Health Policy” OR “Healthcare Policy” OR “Healthcare Policies” OR “Health Policies” OR “Health Care Policies” OR “Health Care Policy” OR “National Health Policy” OR “National Health Policies” OR “public health” OR “Healthcare Economics and Organizations” OR “Health Care Economics” OR “Health Care Economic” OR “Healthcare Economics” OR “Healthcare Economic” OR “Health Economics” OR “Health Economic”) AND (“Propensity Score” OR “Propensity Scores” OR “Inverse Propensity Weighting”)

Database: IEEE Xplore

Search query:

(“All Metadata”:“Machine Learning” OR “All Metadata”:“Transfer Learning” OR “All Metadata”:“Artificial Intelligence” OR “All Metadata”:“Deep Learning” OR “All Metadata”:“cart” OR “All Metadata”:“classification and regression tree” OR “All Metadata”:“gradient boosting machine” OR “All Metadata”:“xgboost” OR “All Metadata”:“Computer Neural Network” OR “All Metadata”:“Computer Neural Networks” OR “All Metadata”:“Neural Networks” OR “All Metadata”:“Neural Network” OR “All Metadata”:“Random Forest” OR “All Metadata”:“Random Forests” OR “All Metadata”:“logistic regression” OR “All Metadata”:“decision tree”) AND (“All Metadata”:“Health Policy” OR “All Metadata”:“Healthcare Policy” OR “All Metadata”:“Healthcare Policies” OR “All Metadata”:“Health Policies” OR “All Metadata”:“Health Care Policies” OR “All Metadata”:“Health Care Policy” OR “All Metadata”:“National Health Policy” OR “All Metadata”:“National Health Policies” OR “All Metadata”:“public health” OR “All Metadata”:“Healthcare Economics and Organizations” OR “All Metadata”:“Health Care Economics” OR “All Metadata”:“Health Care Economic” OR “All Metadata”:“Healthcare Economics” OR “All Metadata”:“Healthcare Economic” OR “All Metadata”:“Health Economics” OR “All Metadata”:“Health Economic”) AND (“All Metadata”:“Propensity Score” OR “All Metadata”:“Propensity Scores” OR “All Metadata”:“Inverse Propensity Weighting”)

Database: Scopus (Elsevier)

Search query:

TITLE-ABS-KEY(“Machine Learning” OR “Transfer Learning” OR “Artificial Intelligence” OR “Deep Learning” OR “cart” OR “classification and regression tree” OR “gradient boosting machine” OR “xgboost” OR “Computer Neural Network” OR “Computer Neural Networks” OR “Neural Networks” OR “Neural Network” OR “Random Forest” OR “Random Forests” OR “logistic regression” OR “decision tree”) AND TITLE-ABS-KEY(“Health Policy” OR “Healthcare Policy” OR “Healthcare Policies” OR “Health Policies” OR “Health Care Policies” OR “Health Care Policy” OR “National Health Policy” OR “National Health Policies” OR “public health” OR “Healthcare Economics and Organizations” OR “Health Care Economics” OR “Health Care Economic” OR “Healthcare Economics” OR “Healthcare Economic” OR “Health Economics” OR “Health Economic”) AND TITLE-ABS-KEY(“Propensity Score” OR “Propensity Scores” OR “Inverse Propensity Weighting”)

Database: Web of Science (Clarivate Analytics)

Search query:

TS = (“Machine Learning” OR “Transfer Learning” OR “Artificial Intelligence” OR “Deep Learning” OR “cart” OR “classification and regression tree” OR “gradient boosting machine” OR “xgboost” OR “Computer Neural Network” OR “Computer Neural Networks” OR “Neural Networks” OR “Neural Network” OR “Random Forest” OR “Random Forests” OR “logistic regression” OR “decision tree”) AND TS = (“Health Policy” OR “Healthcare Policy” OR “Healthcare Policies” OR “Health Policies” OR “Health Care Policies” OR “Health Care Policy” OR “National Health Policy” OR “National Health Policies” OR “public health” OR “Healthcare Economics and Organizations” OR “Health Care Economics” OR “Health Care Economic” OR “Healthcare Economics” OR “Healthcare Economic” OR “Health Economics” OR “Health Economic”) AND TS = (“Propensity Score” OR “Propensity Scores” OR “Inverse Propensity Weighting”)

Database: COMPENDEX

Search query:

(((“Machine Learning” OR “Transfer Learning” OR “Artificial Intelligence” OR “Deep Learning” OR “cart” OR “classification and regression tree” OR “gradient boosting machine” OR “xgboost” OR “Computer Neural Network” OR “Computer Neural Networks” OR “Neural Networks” OR “Neural Network” OR “Random Forest” OR “Random Forests” OR “logistic regression” OR “decision tree”) WN KY) AND ((“Health Policy” OR “Healthcare Policy” OR “Healthcare Policies” OR “Health Policies” OR “Health Care Policies” OR “Health Care Policy” OR “National Health Policy” OR “National Health Policies” OR “public health” OR “Healthcare Economics and Organizations” OR “Health Care Economics” OR “Health Care Economic” OR “Healthcare Economics” OR “Healthcare Economic” OR “Health Economics” OR “Health Economic”) WN KY)) AND ((“Propensity Score” OR “Propensity Scores” OR “Inverse Propensity Weighting”) WN KY)

Database: LILACS

Search query:

(“Machine Learning” OR “Transfer Learning” OR “Artificial Intelligence” OR “Deep Learning” OR “cart” OR “classification and regression tree” OR “gradient boosting machine” OR “xgboost” OR “Computer Neural Network” OR “Computer Neural Networks” OR “Neural Networks” OR “Neural Network” OR “Random Forest” OR “Random Forests” OR “logistic regression” OR “decision tree” OR “Aprendizado de Máquina” OR “Aprendizado Automático” OR “Aprendizado de Transferência” OR “Aprendizagem Automática” OR “Aprendizagem de Máquina” OR “Aprendizagem de Transferência” OR “Aprendizaje Automático” OR “Aprendizaje por Transferencia” OR “Inteligencia Artificial” OR “Aquisição de Conhecimento” OR “Aquisição de Conhecimentos” OR “IA” OR “Inteligência Artificial” OR “Inteligencia de Máquina” OR “Raciocínio Automático” OR “Raciocínio Computacional” OR “Representação de Conhecimento” OR “Representação do Conhecimento” OR “Sistemas de Visão Artificial” OR “Sistemas de Visão Computacional” OR “Adquisición de Conocimientos” OR “Adquisición de Conocimientos” OR “Adquisición de Conocimientosor” OR “Razonamiento Automático” OR “Razonamiento Computacional” OR “Representación del Conocimiento” OR “Sistemas de Visión Artificial” OR “Sistemas de Visión Computacional” OR “Sistemas de Visión por Computador” OR “Sistemas de Visión por Computadora” OR “Sistemas de Visión por Ordenador” OR “Aprendizado Profundo” OR “Aprendizado Estruturado Profundo” OR “Aprendizado Hierárquico” OR “Aprendizaje Profundo” OR “Aprendizaje Jerarquizado” OR “aprendizaje jerárquico” OR “Redes Neurais de Computação” OR “Modelos Conexionistas” OR “Modelos de Rede Neural” OR “Modelos de Redes Neurais” OR perceptrons OR “Redes Neurais” OR “Redes Neuronais em Informática” OR “Redes Neurales de la Computación” OR “Modelos de Conexión” OR “Modelos de Redes Neurales” OR “Modelos de Redes Neuronales” OR perceptrones OR “Redes Neurales” OR “Redes Neuronales Computacionales” OR “redes neuronales informáticas” OR “Algoritmo Florestas Aleatórias” OR “Algoritmo Floresta Aleatória” OR “Classificação de Floresta Aleatória” OR “Floresta Aleatória” OR “Bosques Aleatorios”) AND (“Health Policy” OR “Healthcare Policy” OR “Healthcare Policies” OR “Health Policies” OR “Health Care Policies” OR “Health Care Policy” OR “National Health Policy” OR “National Health Policies” OR “public health” OR “Healthcare Economics and Organizations” OR “Health Care Economics” OR “Health Care Economic” OR “Healthcare Economics” OR “Healthcare Economic” OR “Health Economics” OR “Health Economic” OR “Política de Saúde” OR “Diretrizes das Políticas” OR “Organização Governamental e Políticas” OR “Organização e Políticas Governamentais” OR “PPS Políticas Públicas em Saúde” OR “Plano Nacional de Saúde” OR “Política Nacional de Atenção à Saúde do Homem” OR “Política Nacional de Promoção da Saúde” OR “Política Nacional de Saúde” OR “Política Nacional de Saúde do Homem” OR “Política Nacional de Saúde do Idoso” OR “Política Pública de Saúde” OR “Política Sanitária” OR “Política de Assistência à Saúde” OR “Política de Atenção à Saúde” OR “Política de Saúde Pública” OR “Política em Saúde Pública” OR “Políticas Públicas Saudáveis” OR “Políticas Públicas de Saúde” OR “Políticas Públicas em Saúde” OR “Políticas Sanitárias” OR “Políticas de Cuidados de Saúde” OR “Políticas de Cuidados em Saúde” OR “Políticas de Saúde” OR “Políticas de Saúde Pública” OR “Políticas em Saúde Pública” OR “Política de Salud” OR “Organización Gubernamental y Políticas” OR “Organización y Políticas Gubernamentales” OR “Política Nacional de Salud” OR “Política Pública de Salud” OR “Política Sanitaria” OR “Política de Salud Pública Política en Salud Pública” OR “Políticas Públicas Saludables” OR “Políticas Públicas de Salud” OR “Políticas Públicas en Salud” OR “Políticas Sanitarias” OR “Políticas de Atención en Salud” OR “Políticas de Salud” OR “Políticas de Salud Pública” OR “Políticas en Salud Pública” OR “Economia e Organizações de Saúde” OR “Aspectos Econômicos da Prestação de Serviços de Saúde” OR “Economia da Assistência à Saúde” OR “Economia da Atenção à Saúde” OR “Economia da Saúde” OR “Economia dos Cuidados de Saúde” OR “Economía y Organizaciones para la Atención de la Salud” OR “Economía y Organizaciones para la Atención de la Salud” OR “Economía Sanitaria” OR “Economía de la Atención Médica” OR “Economía de la Salud” OR “Economía en Atención de Salud y Organizaciones”) AND (“Propensity Score” OR “Propensity Scores” OR “Inverse Propensity Weighting” OR “Pontuação de Propensão” OR “Escore de Propensão” OR “Índice de Propensão” OR “Puntaje de Propensión” OR “puntuación de propensión” OR “escala de propensión” OR “puntuación de la propensión”) AND (db:(“LILACS”))

Database: ProQuest Dissertations & Theses Global (PQDT Global)

Search query:

noft(“Machine Learning” OR “Transfer Learning” OR “Artificial Intelligence” OR “Deep Learning” OR “cart” OR “classification and regression tree” OR “gradient boosting machine” OR “xgboost” OR “Computer Neural Network” OR “Computer Neural Networks” OR “Neural Networks” OR “Neural Network” OR “Random Forest” OR “Random Forests” OR “logistic regression” OR “decision tree”) AND noft(“Health Policy” OR “Healthcare Policy” OR “Healthcare Policies” OR “Health Policies” OR “Health Care Policies” OR “Health Care Policy” OR “National Health Policy” OR “National Health Policies” OR “public health” OR “Healthcare Economics and Organizations” OR “Health Care Economics” OR “Health Care Economic” OR “Healthcare Economics” OR “Healthcare Economic” OR “Health Economics” OR “Health Economic”) AND noft(“Propensity Score” OR “Propensity Scores” OR “Inverse Propensity Weighting”)

Database: Scholar Google

Search query:

(“Machine Learning” OR “Artificial Intelligence” OR “Neural Network” OR “Random Forest” OR “logistic regression”) AND (“Health Policy” OR “Healthcare Policy” OR “Health Economic”) AND (“Propensity Score” OR “Inverse Propensity Weighting”)

## References

[1] Morgenstern J.D., Buajitti E., O’Neill M., Piggott T., Goel V., Fridman D., Kornas K., Rosella L.C. (2020). Predicting Population Health with Machine Learning: A Scoping Review. BMJ Open.

[2] Kreif N., DiazOrdaz K. (2019). Machine Learning in Policy Evaluation: New Tools for Causal Inference. Oxford Research Encyclopedia of Economics and Finance.

[3] Stuart E.A. (2010). Matching Methods for Causal Inference: A Review and a Look Forward. Stat. Sci..

[4] Athey S., Imbens G.W. (2017). The State of Applied Econometrics: Causality and Policy Evaluation. J. Econ. Perspect..

[5] Conrady S., Jouffe L., Elwert F. (2014). Causality for Policy Assessment and Impact Analysis—Directed Acyclic Graphs and Bayesian Networks for Causal Identification and Estimation.

[6] Cannas M., Arpino B. (2019). A Comparison of Machine Learning Algorithms and Covariate Balance Measures for Propensity Score Matching and Weighting. Biom. J..

[7] Rodrigues D., Kreif N., Lawrence-Jones A., Barahona M., Mayer E. (2022). Reflection on Modern Methods: Constructing Directed Acyclic Graphs (DAGs) with Domain Experts for Health Services Research. Int. J. Epidemiol..

[8] Lin L., Zhu Y., Chen L. (2019). Causal Inference for Multi-Level Treatments with Machine-Learned Propensity Scores. Health Serv. Outcomes Res. Methodol..

[9] Mooney S.J., Pejaver V. (2018). Big Data in Public Health: Terminology, Machine Learning, and Privacy. Annu. Rev. Public Health.

[10] Elze M.C., Gregson J., Baber U., Williamson E., Sartori S., Mehran R., Nichols M., Stone G.W., Pocock S.J. (2017). Comparison of Propensity Score Methods and Covariate Adjustment: Evaluation in 4 Cardiovascular Studies. J. Am. Coll. Cardiol..

[11] Mokhles S., Takkenberg J.J., Treasure T. (2017). Evidence-Based and Personalized Medicine. It’s [AND] Not [OR]. Ann. Thorac. Surg..

[12] Westreich D., Lessler J., Funk M.J. (2010). Propensity Score Estimation: Neural Networks, Support Vector Machines, Decision Trees (CART), and Meta-Classifiers as Alternatives to Logistic Regression. J. Clin. Epidemiol..

[13] Carter D.J., Daniel R., Torrens A.W., Sanchez M.N., Maciel E.L.N., Bartholomay P., Barreira D.C., Rasella D., Barreto M.L., Rodrigues L.C. (2019). The Impact of a Cash Transfer Programme on Tuberculosis Treatment Success Rate: A Quasi-Experimental Study in Brazil. BMJ Glob. Health.

[14] Ahmed S., Sarker A.R., Sultana M., Chakrovorty S., Ahmed M.W., Dorin F., Mirelman A.J., Islam Z., Rahman M.H., Niessen L.W. (2018). The Impact of Community-Based Health Insurance on the Utilization of Medically Trained Healthcare Providers among Informal Workers in Bangladesh. PLoS ONE.

[15] Shen C.T., Chen F.M., Hsieh H.M. (2020). Effect of a National Population-Based Breast Cancer Screening Policy on Participation in Mammography and Stage at Breast Cancer Diagnosis in Taiwan. Health Policy.

[16] Lee B.K., Lessler J., Stuart E.A. (2010). Improving Propensity Score Weighting Using Machine Learning. Stat. Med..

[17] Cheng L., Guo R., Moraffah R., Sheth P., Candan K.S., Liu H. (2022). Evaluation Methods and Measures for Causal Learning Algorithms. IEEE Trans. Artif. Intell..

[18] Ashrafian H., Darzi A. (2018). Transforming Health Policy through Machine Learning. PLoS Med..

[19] Kühne F., Schomaker M., Stojkov I., Jahn B., Conrads-Frank A., Siebert S., Sroczynski G., Puntscher S., Schmid D., Schnell-Inderst P. (2022). Causal Evidence in Health Decision Making: Methodological Approaches of Causal Inference and Health Decision Science. GMS Ger. Med. Sci..

[20] Tricco A.C., Lillie E., Zarin W., O’Brien K.K., Colquhoun H., Levac D., Moher D., Peters M.D.J., Horsley T., Weeks L. (2018). PRISMA Extension for Scoping Reviews (PRISMA-ScR): Checklist and Explanation. Ann. Intern. Med..

[21] Chen H., Xing J., Yang X., Zhan K. (2021). Heterogeneous Effects of Health Insurance on Rural Children’s Health in China: A Causal Machine Learning Approach. Int. J. Environ. Res. Public Health.

[22] Costello M.J., Li Y., Zhu Y., Walji A., Sousa S., Remers S., Chorny Y., Rush B., MacKillop J. (2021). Using Conventional and Machine Learning Propensity Score Methods to Examine the Effectiveness of 12-Step Group Involvement Following Inpatient Addiction Treatment. Drug Alcohol Depend..

[23] Ladhania R., Haviland A.M., Venkat A., Telang R., Pines J.M. (2021). The Effect of Medicaid Expansion on the Nature of New Enrollees’ Emergency Department Use. Med. Care Res. Rev..

[24] Maciel F.A., Duarte D. (2023). The Impact of Cash Transfer Participation on Unhealthy Consumption in Brazil. Health Policy Open.

[25] Kreif N., DiazOrdaz K., Moreno-Serra R., Mirelman A., Hidayat T., Suhrcke M. (2022). Estimating Heterogeneous Policy Impacts Using Causal Machine Learning: A Case Study of Health Insurance Reform in Indonesia. Health Serv. Outcomes Res. Methodol..

[26] Wink Junior M.V., dos Santos F.L., Hoffmann M.G., Garcia L.P. (2022). Impact Assessment of Emergency Care Units on Hospitalizations for Respiratory System Diseases in Brazil. Cienc. Saude Colet..

[27] Garcia L.P., Schneider I.J.C., de Oliveira C., Traebert E., Traebert J. (2023). What Is the Impact of National Public Expenditure and Its Allocation on Neonatal and Child Mortality? A Machine Learning Analysis. BMC Public Health.

[28] Padula W.V., Kreif N., Vanness D.J., Adamson B., Rueda J.-D., Felizzi F., Jonsson P., IJzerman M.J., Butte A., Crown W. (2022). Machine Learning Methods in Health Economics and Outcomes Research—The PALISADE Checklist: A Good Practices Report of an ISPOR Task Force. Value Health.

[29] VanderWeele T.J., Ding P. (2017). Sensitivity Analysis in Observational Research: Introducing the E-Value. Ann. Intern. Med..

[30] Liu W., Kuramoto S.J., Stuart E.A. (2013). An Introduction to Sensitivity Analysis for Unobserved Confounding in Nonexperimental Prevention Research. Prev. Sci..

